# Nutrients Bioaccessibility and Anti-inflammatory Features of Fermented Bee Pollen: A Comprehensive Investigation

**DOI:** 10.3389/fmicb.2021.622091

**Published:** 2021-02-02

**Authors:** Pasquale Filannino, Raffaella Di Cagno, Olimpia Vincentini, Daniela Pinto, Andrea Polo, Francesca Maialetti, Annalisa Porrelli, Marco Gobbetti

**Affiliations:** ^1^Department of Soil, Plant and Food Science, University of Bari Aldo Moro, Bari, Italy; ^2^Faculty of Sciences and Technology, Libera Università di Bolzano, Bolzano, Italy; ^3^Unit of Human Nutrition and Health, Department of Food Safety, Nutrition and Veterinary Public Health, Istituto Superiore di Sanità, Rome, Italy; ^4^Giuliani S.p.A., Milan, Italy

**Keywords:** fructophilic lactic acid bacteria, fermentation, starter culture, bee bread, bee-collected pollen, anti-inflammatory

## Abstract

We compared raw bee-collected pollen (Raw-BCP), spontaneously fermented BCP (Unstarted-BCP), and BCP fermented with selected microbial starters (Started-BCP) to deepen whether fermentation may favorably affect the nutrients bioaccessibility and functional features of BCP. Under *in vitro* gastrointestinal batches, the highest serum-availability of phenolic compounds was found in Started-BCP, highlighting the positive effect exerted by selected microbial starters. The same effect was not found in spontaneously fermented BCP. In colon adenocarcinoma cell line-2 (Caco-2) cells stressed by a pro-inflammatory stimulus, the treatment with Started-BCP halted the increase of pro-inflammatory mediator’s level. Started-BCP counteracted efficiently the deleterious effects of inflammatory stimuli on the integrity of the Caco-2 cells monolayer and its barrier function. Started-BCP successfully counteracted the H_2_O_2_-induced intracellular accumulation of reactive oxygen species (ROS) in Caco-2 cells. A protective role against lipopolysaccharide (LPS)-induced inflammation was exerted by Started-BCP in human keratinocytes. The same protective effects on Caco-2 and keratinocyte cell lines were negligible after treatments with Raw-BCP or Unstarted-BCP.

## Introduction

Bee bread (BB) and bee-collected pollen (BCP) are recognized as valuable dietary supplements for human nutrition, due to their appreciable content of proteins, essential amino acids, unsaturated fatty acids, minerals, vitamins, phenolic compounds, carotenoid pigments, and phytosterols (Khalifa et al., 2020; Thakur and Nanda, 2020). This plethora of nutrients and functional compounds mediate a variety of biological effects, such as antiradicals, anticancer, anti-inflammatory, hepatoprotective, anti-atherosclerotic, and immunomodulatory (Khalifa et al., 2020; Thakur and Nanda, 2020). Recently, the potential of bee pollen to restore impaired intestinal barrier function gained the attention of researchers (Chen et al., 2019; Li et al., 2019). Due to a rising awareness of chronic inflammatory disorders of the gastrointestinal tract as one of the major chronic diseases worldwide, seeking out novel effective nutritional approaches without side effects became an attracting topic (Di Cagno et al., 2019b).

Bee bread results by the mixture of plant pollen, nectar, honey, and bees-glandular secretions, which is packed by honeybees in hive cells and undergo a maturation process mediated by bee-associated microbial communities (Anderson et al., 2011). Although several technologies have been proposed for the collection of BB from honeycombs, all these methods suffer from limitations (e.g., nutrient loss, labor intensiveness, and detrimental effect to the hive; Urcan et al., 2017). For this reason, BB marketing is unexploited by most of beekeepers. Conversely, BCP is collected before undergoing any maturation process by traps fixed at the entrance of beehives, resulting as convenient technology for beekeepers and harmless to the hive (Campos et al., 2010). BB and BCP are nutritionally and biochemically different, even though bee pollen is the main ingredient of BB. In fact, the outer layer of the grain pollen (intine and exine) is not easily digestible by mono-gastric organisms, such as humans, and may reduce the nutrients bioaccessibility by more than 50% (Kieliszek et al., 2018; Zuluaga-Domínguez et al., 2019; Kostić et al., 2020). The conversion of BCP to BB leads to several biochemical changes, including the modification of the intine-exine complex, which in turn result into increased nutritional value and digestibility (Kieliszek et al., 2018; Kostić et al., 2020). These changes suggest that BCP needs to be processed before the human consumption to increase the availability of nutrients for intestinal absorption. One of the most attractive options is the simulation of the natural fermentation occurring within the hive leading to the BB (Zuluaga et al., 2014). Recently, we developed a fermentation protocol of BCP, based on the fermentation with a selected consortium composed by *Apilactobacillus kunkeei* strains and *Hanseniaspora uvarum*, which led to stable, safe, and standardized fermented product with increased protein digestibility and free phenolics concentration (Di Cagno et al., 2019a). BCP represents an extreme environment for bacteria due to the low pH value, osmotic stress, and the high level of phenolics, where *A. kunkeei* resulted a well-adapted species (Di Cagno et al., 2019a; Filannino et al., 2019).

The prominent role of microbes in promoting the nutrient bioavailability during BCP fermentation is debated (Anderson et al., 2014; Carroll et al., 2017; Di Cagno et al., 2019a), and few intervention studies on fermented BCP intake are available (Uțoiu et al., 2018; Bakour et al., 2019; Kostić et al., 2020). Under the conditions of our study, we compared fermented and unfermented BCP in a systematic way to deepen whether fermentation may affect the nutrients bioaccessibility, and the anti-inflammatory and immunomodulatory properties of BCP. *In vitro* and *ex vivo* models closest to the *in vivo* systems were used. Under the *in vitro* gastrointestinal batch, we provided original evidence about the availability of phenolics after simulated digestion of fermented BCP. Although BCP represents a valuable reservoir of phenolic compounds, which are receiving attention for their wide range of health-promoting functions (Li et al., 2018), their bioaccessibility represents a critical point for using BCP as dietary supplement (Kieliszek et al., 2018; Zuluaga-Domínguez et al., 2019; Kostić et al., 2020). The ability of fermented BCP to modify the cellular redox status [production of reactive oxygen species (ROS)], to modulate the secretion of pro-inflammatory mediators [interleukin-8 (IL-8), interleukin-6 (IL-6), monocyte chemotactic protein-1 (MCP-1), tumor necrosis factors-*α* (TNF-α), and prostaglandin E2 (PGE-2)], and to counteract the disruption of epithelial integrity was investigated through a model of intestinal absorption using the human colon adenocarcinoma cell line-2 (Caco-2). After confluence, Caco-2 cells differentiate structurally and functionally into enterocyte-like representing a suitable model to assess the physiological response of intestinal mucosa to oxidative stress and inflammatory status (Bedoya-Ramírez et al., 2017; Di Cagno et al., 2019b). A growing amount of studies confirmed the role of postbiotics as promoters of a general health state, including dermatological level (Knackstedt et al., 2020; Rinaldi et al., 2020). Thus, the anti-inflammatory activity of fermented BCP was investigated using keratinocytes as additional model (Lagha and Grenier, 2019; Sánchez-Marzo et al., 2019). Keratinocytes are actively involved in the cutaneous immune responses through the expression of cytokines and chemotactic factors, which can transmit both positive and negative signals to cells of innate and adaptive immunity (Albanesi et al., 2005).

## Materials and Methods

### Microorganisms and Culture Conditions

*A. kunkeei* PF12, PL13, and PF15 and *H. uvarum* AN8Y27B belonging to the Culture Collection of the Department of Soil, Plant and Food Science, University of Bari Aldo Moro (Bari, Italy), were used as mixed starters for BCP fermentation. Their aptitude to drive standardized fermentation of BCP at laboratory, pilot-plant, and full-scale levels was preliminarily verified (Di Cagno et al., 2019a; Giuliani et al., 2020). Cultures were maintained as stocks in 15% (v v^−1^) glycerol at −80°C and routinely propagated. Fructophilic lactic acid bacteria were cultured at 30°C for 24 h in fructose-yeast extract-polypeptone (FYP) broth (10 g D-fructose, 10 g yeast extract, 5 g polypeptone, 2 g sodium acetate, 0.5 g Tween 80, 0.2 g MgSO_4_·7H_2_O, 0.01 g MnSO_4_·4H_2_O, 0.01 g FeSO_4_·7H_2_O, and 0.01 g NaCl per liter of distilled water). *H. uvarum* AN8Y27B was cultivated at 30°C for 36 h in yeast extract-peptone-dextrose (YPD) broth (10 g yeast extract, 20 g bacteriological peptone, and 20 g dextrose per liter of distilled water).

### Fermentation of BCP

Bee-collected ivy pollen (BCP) was collected during September–October 2018 from organic fields of the Apulia region (Italy). BCP was fermented under a standardized protocol previously described by Di Cagno et al. (2019a), which included a mixed inoculum of *A. kunkeei* PF12, PL13, and PF15 strains, and *H. uvarum* AN8Y27B. Briefly, microorganisms were cultivated until the late exponential growth phase was reached, washed twice in 50 mM phosphate buffer (pH 7.0), and used to inoculate the BCP at the final density of ca. 8 Log CFU g^−1^. BCP was added with sterile water to reach the final water content of 40% (w w^−1^), inoculated with the mixed starter, placed into sealed tubes, and incubated at 30°C for 216 h. BCP fermented by the selected mixed starter (Started-BCP) was characterized as described below. BCP treated under the same conditions except for the use of microbial starters (Unstarted-BCP), and fresh BCP without any treatment [raw bee-collected pollen (Raw-BCP)] were used as controls. Unstarted-BCP underwent spontaneous fermentation to partially mimic the spontaneous fermentation of beebread within hive cells. Mesophilic lactic acid bacteria and yeast cell densities were monitored through plate counts on FYP agar containing 0.1% of cycloheximide (Sigma Aldrich, Saint Louis, MO, United States) incubated at 30°C for 48 h, and on YPD agar added of 0.1% of chloramphenicol incubated at 25°C for 72 h, respectively. Freeze-dried BCP samples were used for the subsequent assays.

### *In vitro* Gastrointestinal Batch Digestion and Phenolics Bioaccessibility Assay

To evaluate the nutrients and bioactive compounds bioaccessibility in Raw-, Unstarted-, and Started-BCP, we chose phenolics as target compounds. Phenolics bioaccessibility in BCP samples was investigated through an *in vitro* gastrointestinal batch digestion process according to Eid et al. (2014) and Celep et al. (2015), with few modifications. BCP (10 g) was homogenized in distilled water (50 ml) for 2 min using a stomacher. Then, 20 mg of α-amylase were dissolved in 6.25 ml of CaCl_2_ (1 mM) solution and added to the mixture. The mixture was incubated at 37°C for 30 min under stirring condition (100 rpm) to mimic the oral digestion phase. To simulate gastric digestion, pepsin (2.7 g) was dissolved in 25 ml of 0.1 M HCl and added to mixture. Then, the pH value was adjusted to 2.0 using 6 M HCl and the mixture was incubated at 37°C for 3 h under stirring condition. To simulate small intestine conditions, pancreatin (560 mg) and bile (3.5 g) were dissolved in 125 ml of 0.1 M NaHCO_3_ and added to the mixture. Value of pH was slowly adjusted to 7.0 by using 6 M NaOH and a segment of cellulose dialysis tubing (molecular weight cut off 12 kDa) was placed inside the beaker. The mixture was incubated at 37°C for 3 h under stirring condition (100 rpm). After the incubation, the solution that diffused into the dialysis tubing was taken as the serum-available fraction. The latter was centrifuged at 16,000 rpm, filtered through a nylon syringe filter with a pore size of 0.45 μm, and total phenolic compounds were assayed according to Folin-Ciocalteu method (Singleton and Rossi, 1965; Singleton et al., 1999). Data were expressed as gallic acid g equivalents per liter of digested BCP. *In vitro* digested BCP samples were freeze-dried and used for the subsequent assays.

### Cultures of Human Caco-2 Cells

Human Caco-2 cells were obtained from ATCC (ATCC® HTB-37™) and were used between passages 15 and 35 for all experiments. Cells were cultured in high glucose DMEM supplemented with 10% (v v^−1^) Fetal Bovine Serum (FBS), 1% (v v^−1^) HEPES, NEAA, and 1% (v v^−1^) penicillin/streptomycin, maintained at 37°C in a 5% CO_2_ incubator, and were sub-cultured at 80–90% confluence every 3–4 days.

### Caco-2 Cells Viability Assay

Colon adenocarcinoma cell line-2 cells metabolic activity was assessed through the 3-(4,5-dimethylthiazol-2-yl)-2,5-diphenyltetrazolium bromide (MTT) assay. Cells were seeded at a concentration of 2 × 10^4^ in a 96-well plate. After confluence was reached, cells were treated with Raw-, Unstarted-, and Started-BCP at different concentrations (10–500 μg ml^−1^) for 24 h. At the end of the treatment exposure, medium was discarded and replaced by 100 μl of 0.5 mg ml^−1^ MTT solution. After 2 h of incubation at 37°C, the MTT solution was discarded, and formazan crystals were dissolved in 100 μl dimethyl sulfoxide, and absorbance was read in a multiwall plate reader (Bio-Rad, Hercules, CA, United States) at 570 nm. Percent viability was calculated relative to untreated Caco-2 cells.

### LDH Release in Caco-2 Cells

Cytotoxicity of Raw-, Unstarted-, and Started-BCP was determined by measuring the release of lactate dehydrogenase (LDH) into the Caco-2 culture medium. Caco-2 cells were seeded in a 96-well plate at a density of 1.5 × 10^4^ cells/well in 200 μl medium. On the fifth day of culture, the cells were treated with various concentrations of RW pollen or fermented pollen. After 24 h incubation, supernatants were collected and centrifuged at 200 × *g* for 5 min at room temperature and assayed using Pierce LDH cytotoxicity assay kit (Thermo Fisher Scientific, Waltham, MA, United States) according to the manufacturer’s instructions to evaluate LDH concentration in the culture medium. Percent cytotoxicity was calculated as follows:

$$\%\;\mathrm{Cytotoxicity}\;=\;\left[\begin{array}{l} \left(\begin{array}{l} \mathrm{BCP}\;-\;\mathrm{treated}\;\mathrm{LDH}\;\mathrm{activity}\;-\;\\ \mathrm{Spontaneous}\;\mathrm{LDH}\;\mathrm{activity} \end{array}\right)/\\ \left(\begin{array}{l} \mathrm{Maximum}\;\mathrm{LDH}\;\mathrm{activity}\;-\;\\ \mathrm{Spontaneous}\;\mathrm{LDH}\;\mathrm{activity} \end{array}\right) \end{array}\right]\;\times \;100$$

where untreated cells are referred to as Spontaneous LDH Activity Control and 5% triton x-100 treated cells are referred to as Maximum LDH Activity Control.

### Release of Pro-inflammatory Mediators by Caco-2 Cells

Interleukin-8, IL-6, MCP-1, TNF-α, and PGE-2 release by Caco-2 cells after 24 h of treatment with Raw-, Unstarted-, and Started-BCP (100 μg ml^−1^) was quantified using commercial ELISA kits. Caco-2 cells pre-treated with BCP for 6 h were further stimulated with an inflammatory stimulus and then incubated for others 18 h. The inflammatory stimulus for IL-8, IL-6, MCP-1, and TNF-α was the interleukin-1β (IL-1β) at the concentration of 25 ng ml^−1^. A cytokines mix (LPS, 10 ng ml^−1^; TNF-α, 50 ng ml^−1^; and IL-1β, 25 ng ml^−1^) was the inflammatory stimulus for PGE-2. ELISA kit (R&D Systems) were used according to manufacturer’s instructions. Optical density was read with a microplate reader (Bio-Rad) at 450 nm.

### Measurement of Reactive Oxygen Species in Caco-2 Cells

Intracellular ROS content was assessed using 2',7'–dichlorodihydrofluorescein diacetate acetyl ester (DCFH-DA; Invitrogen). Cells were incubated with 100 μg ml^−1^ of Raw-, Unstarted-, and Started-BCP for 24 h, and a set of samples was exposed to 50 μM H_2_O_2_ in the last 6 h to induce ROS production. At the end of the treatment, cells were rinsed twice with PBS and incubated with 80 μM DCFH-DA (Life Technologies), prepared in complete cell culture medium, for 30min at 37°C. They were harvested by scraping, and DCFH-DA fluorescence intensity (FI) was measured at *λ*_exc_/λ_em_ 480/570 nm (Agilent Technologies). After background removal (λ_exc_/λ_em_ 480/650 nm), DCF fluorescence was normalized to protein concentration.

### Transepithelial Electrical Resistance

Transepithelial electrical resistance (TEER) was used as a measure of cell monolayer integrity and was assessed before and after all treatments. Caco-2 cells were seeded at a density of 2 × 10^5^ cells per ml onto polycarbonate membrane Transwell inserts with 0.4 μm pore size (Corning, Inc.; Lowell, MA). Cells were cultured for 21 days to reach differentiation, and growth media were refreshed every 2–3 days. Fully differentiated monolayers showed TEER values of 500–700 Ohms. Differentiated Caco-2 monolayers were treated with Raw-, Unstarted-, and Started-BCP at a concentration of 100 μg ml^−1^ for 24 h, and a set of samples was exposed to a mix of inflammatory cytokines in the last 18 h to mimic chronic intestinal barrier dysfunction associated with inflammation. Cytomix consisted in IL-1β (25 ng ml^−1^), TNF-α (50 ng ml^−1^), and IFN-γ (50 ng ml^−1^). At the end of the experiment, plates were then transferred to a Thermoplate set at 37°C and TEER was measured using an epithelial volt-ohm meter with a chopstick electrode (Millicell ERS-2, EMD Millipore, Billerica, MA). The electrode was immersed at a 90° angle with one tip in the basolateral chamber and the other in the apical chamber. Care was taken to avoid electrode contact with the monolayer. An insert without cells was used as a blank and its mean resistance was subtracted from all samples. TEER was expressed as Ohms × cm^2^.

### Permeability Assay

Fluorescein isothiocyanate-dextran (FITC-dextran; MW 4 kD; Sigma Aldrich) was used as a paracellular marker for Caco-2 cell monolayers. At the end of 24 h of TEER measurement, cells were washed with PBS and 1 mg ml^−1^ of FITC-dextran in PBS was added to the apical side of the cell monolayer, and in the basolateral compartment only PBS. Two hundred microliters of samples were collected from the basolateral compartment 2 h later and transferred into 96-well plates, and the diffused fluorescent tracer was measured by fluorometry (*λ*_exc_/λ_em_ 485/528 nm). The intensity of FITC-dextran fluorescence was measured by a fluorescence spectrophotometer (Agilent Technologies, Santa Clara, CA, United States) at excitation/emission of 495/525 nm.

### Cultures of Human Keratinocytes

Normal human keratinocyte NCTC 2544 (National Institute on Cancer Research, Italy) were cultured at 5% CO_2_, 37°C on RPMI medium containing 2 mM l-glutamine, 1% of penicillin (100 U ml^−1^), and streptomycin (100 U ml^−1^), supplemented with 10% FBS (basal medium). Cells were incubated in 25 cm^2^ surface culture flasks at 37°C with 5% CO_2_ until ca. About 80% of confluence was reached. Cells were then harvested with trypsin/EDTA and seeded at a density of 5 × 10^4^ cells per well into 96-well plates for MTT assay and 1 × 10^6^ cells per well into 12-well plates for qRT-PCR, respectively.

### Relative Expression of TNF-α Gene in Human Keratinocytes

Twenty-four hours after seeding on 12-well plates, NCTC2544 80% confluent cells were exposed to Raw-, Unstarted-, and Started-BCP (100 μg ml^−1^) for 16–24 h. A set of samples was simultaneously exposed to LPS 10 μg ml^−1^. RPMI medium with 2.5% FBS, 2 mM l-glutamine, and 1% of penicillin (100 U ml^−1^) and streptomycin (100 U ml^−1^) was used as basal medium. Cells in basal medium were used as negative control and cells incubated only with LPS were used as the positive control. After the medium treatment, RNA for qRT-PCR analysis was extracted. Tri Reagent (Sigma Aldrich) method as described by Chomczynski and Mackey (1995) was used. cDNA was then synthesized from 2 μg RNA template in a 20 μl reaction volume, using the PrimeScript RT-PCR Kit (Takara, Japan). cDNA was amplified and detected by the Stratagene Mx3000P Real-Time PCR System (Agilent Technologies). The amplification of cDNA from NCTC2544 cells was conducted using the following Taqman gene expression assays: Hs00174128_m1 (*TNF-α*) as target gene and Hs999999 m1 [human glyceraldehyde-3-phosphate dehydrogenase (*GAPDH*)] as housekeeping genes, respectively. PCR amplifications were carried out in a 20 μl of total volume. The mixture of reaction contained 10 μl of 2X Premix Ex Taq (Takara), 1 μl of 20× TaqMan gene expression assay, 0.4 μl of RoX Reference Dye II (Takara), 4.6 μl of water, and 4 μl of DNA. PCR conditions were the following: 95°C for 30 s followed by 40 cycles of 95°C for 5 s, 60°C for 20 s. PCR reactions were performed using a MX3000p PCR machine (Stratagene, La Jolla, CA). *Δ* cycle threshold (Vigetti et al., 2008) was used for the calculation of the relative abundance in the expression of each gene.

### Statistical Analysis

Analyses were carried out in triplicate on three biological replicates for each condition. Data were subjected to ANOVA test for multiple comparisons (one-way ANOVA followed by Tukey’s procedure at *p* < 0.05), using the statistical software, Statistica 7.0 (Statsoft).

## Results

### BCP Processing

Started-BCP was inoculated at the final density of ca. 8 Log CFU g^−1^ with the selected mixed starter composed by *A. kunkeei* PF12, PL13, and PF15, and *H. uvarum* AN8Y27B. The aptitude of strains as well the process parameters were previously investigated by Di Cagno et al. (2019a). The initial cell densities of lactic acid bacteria and yeasts in the Unstarted-BCP were 5.41 ± 0.21 and 6.53 ± 0.27 Log CFU g^−1,^ respectively. During fermentation of Started-BCP, the cell density of the lactic bacteria in BCP reached ca. 9 Log CFU g^−1^ after 96 h, remained almost stable until 144 h, then decreased (*p* < 0.05) to 7.15 ± 0.29 Log CFU g^−1^ throughout the incubation time. On the other side, during the spontaneous fermentation of Unstarted-BCP, lactic bacteria reached a cell density of ca. 9 Log CFU g^−1^ only after 120 h, and suddenly decreased (*p* < 0.05) to 4.0 ± 0.31 Log CFU g^−1^. During the first 24 h of incubation, cell density of yeast slightly increased (*p* > 0.05) both in Started- and Unstarted-BCP, then progressively decreased throughout the incubation time until ca. 4 Log CFU g^−1^.

### *In vitro* Gastrointestinal Batch Digestion of BCP and Phenolics Bioaccessibility Assay

Bioaccessibility of nutrients and functional compounds, intended as the fraction of their total amount that is available for human metabolism, represent a critical issue for BCP deserving investigation. This usually entails implementation of *in vitro* simulated gastrointestinal digestion. Following pancreatic digestion and dialysis, the total phenolics in the serum-available fraction obtained from Raw-BCP was 1.67 ± 0.07 g l^−1^, whereas serum-available phenolics from Started-BCP were significantly (*p* < 0.05) higher of ca. 22%. Phenolics availability did not change (*p* > 0.05) in Unstarted-BCP.

### Cytotoxicity Assays on Caco-2 Cells

**Figure 1:**
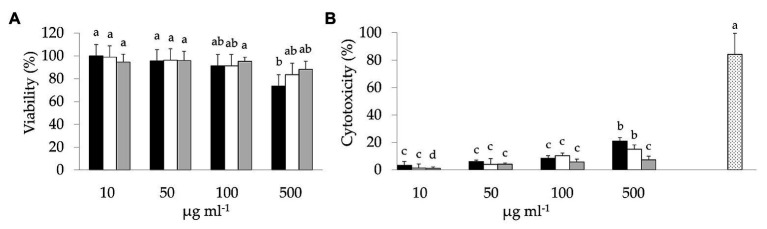
3-(4,5-Dimethylthiazol-2-yl)-2,5-diphenyltetrazolium bromide (MTT) and lactate dehydrogenase (LDH) assays on colon adenocarcinoma cell line-2 (Caco-2) cells: **(A)** Viability (%) of Caco-2 cells treated with different concentrations (10–500 μg ml^−1^) of raw bee-collected pollen (Raw-BCP; black bars), Unstarted-BCP (white), and Started-BCP (gray). Percent viability was determined through the MTT assay and it was calculated respect to untreated Caco-2 cells; **(B)** LDH release (%) into culture medium by the Caco-2 cells treated with different concentrations (10–500 μg ml^−1^) of Raw-BCP (black bars), Unstarted-BCP (white), and Started-BCP (gray). Percent cytotoxicity was calculated as follows: % Cytotoxicity = [(BCP-treated LDH activity – Spontaneous LDH activity)/(Maximum LDH activity – Spontaneous LDH activity)] × 100. Untreated Caco-2 cells are referred to as Spontaneous LDH Activity, whereas 20% triton x-100 treated cells are referred to as Maximum LDH Activity (dotted bar). Data are the means (± SD) of three biological replicates analyzed in triplicate. Bars with different superscript letters are significantly different (*p* < 0.05).

Preliminarily, the cytotoxicity of BCP samples was investigated through the MTT and LDH assays (Figure 1). A slight dose-dependent decrease of cell viability was observed in Caco-2 cells due to the exposure to BCP (Figure 1A). BCP treatment up to 100 μg ml^−1^ showed no cytotoxic effects. Higher dose treatment (500 μg ml^−1^) with Raw-BCP significantly (*p* < 0.05) but slightly impaired Caco-2 cells proliferation, whereas cells viability still approached the control level following the treatment with 500 μg ml^−1^ of Unstarted- and Started-BCP. Regarding the LDH release (Figure 1B), membrane stability strongly decreased following the treatment with 500 μg ml^−1^ of BCP, whereas negligible decreases were detected up to 100 μg ml^−1^. With both 10 and 500 μg ml^−1^ treatments, Started-BCP led to lower LDH release compared to Raw- and Unstarted-BCP. To avoid cytotoxicity from BCP, a concentration of 100 μg ml^−1^ was selected for the subsequent assays on Caco-2 cells as the highest dose without substantial cytotoxic effects.

### Secretion of Pro-inflammatory Mediators by Caco-2 Cells Under BCP Treatments

**Figure 2:**
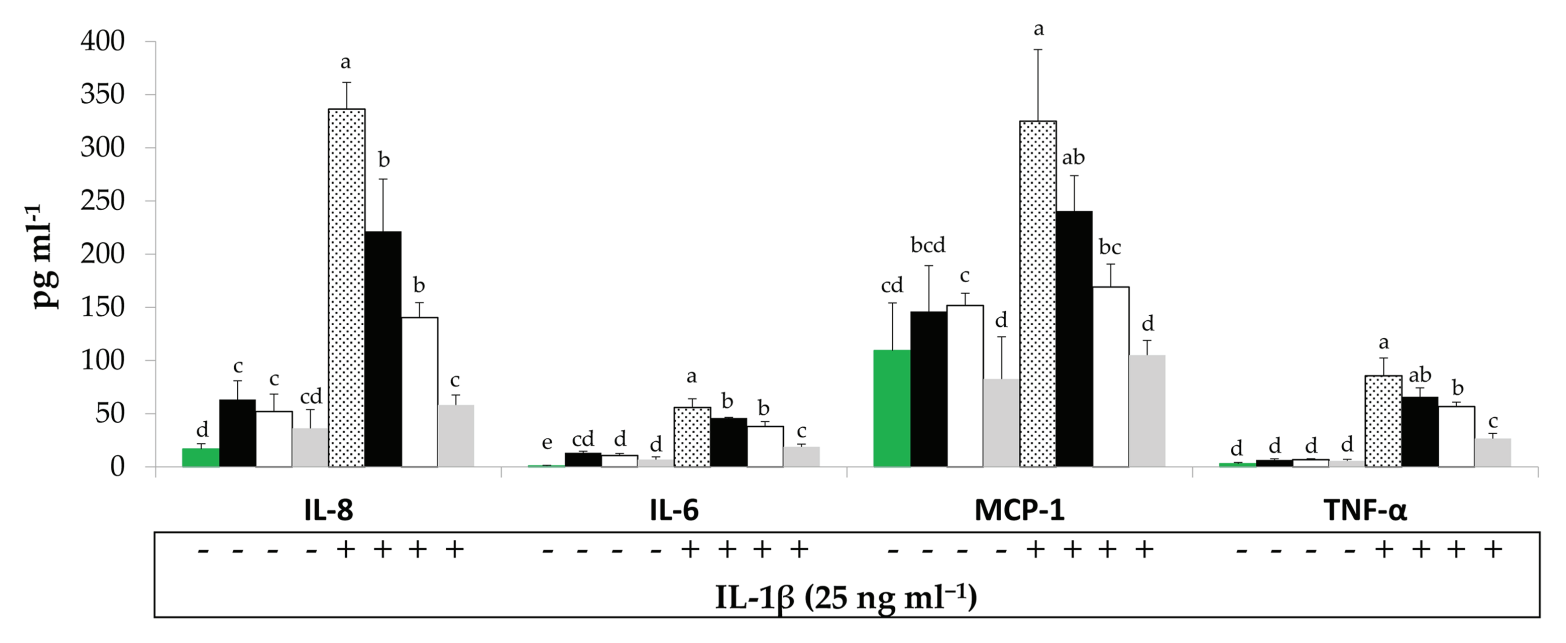
Interleukin-8 (IL-8), interleukin-6 (IL-6), Monocyte chemotactic protein-1 (MCP-1), and Tumor necrosis factors-α (TNF-α) concentration (pg ml^−1^) released by Caco-2 cells. Cells were treated for 24 h with 100 μg ml^−1^ of Raw-BCP (black bars), Unstarted-BCP (white), and Started-BCP (gray). A set of samples was stimulated with the interleukin-1β (IL-1β; 25 ng ml^−1^) in the last 18 h. Levels of cytokines released by untreated cells (negative control, green bars) and by cells treated only with the IL-1β (positive control, dotted bars) were also reported. Data are the means (± SD) of three biological replicates analyzed in triplicate. Bars with different superscript letters are significantly different (*p* < 0.05).

To evaluate potential interactions between BCP treatments and sinthesis of pro-inflammatory mediators by Caco-2 cells, their secretion in the culture medium was quantified by ELISA. Under regular physiological conditions, Raw- and Unstarted-BCP treatments slightly (*p* < 0.05) enhanced the release of IL-8 compared to the untreated Caco-2 cells (negative control; Figure 2). A slight but not significant (*p* > 0.05) increase was induced by the Started-BCP (Figure 2). Further treatment with the pro-inflammatory IL-1β stimulated the secretion of IL-8 compared to the negative control. The effect of the pro-inflammatory stimulus was significantly (*p* < 0.05) counteracted by the Raw- and Unstarted-BCP treatments, and was fully inhibited by the Started-BCP. A similar trend was observed for IL-6 (Figure 2). BCP-treatments lightly induced the IL-6 release under regular physiological conditions, but strongly counteracted the stimulus of IL-1β, with the highest inhibitory effect on IL-6 release observed with the Started-BCP treatment (Figure 2).

**Figure 3:**
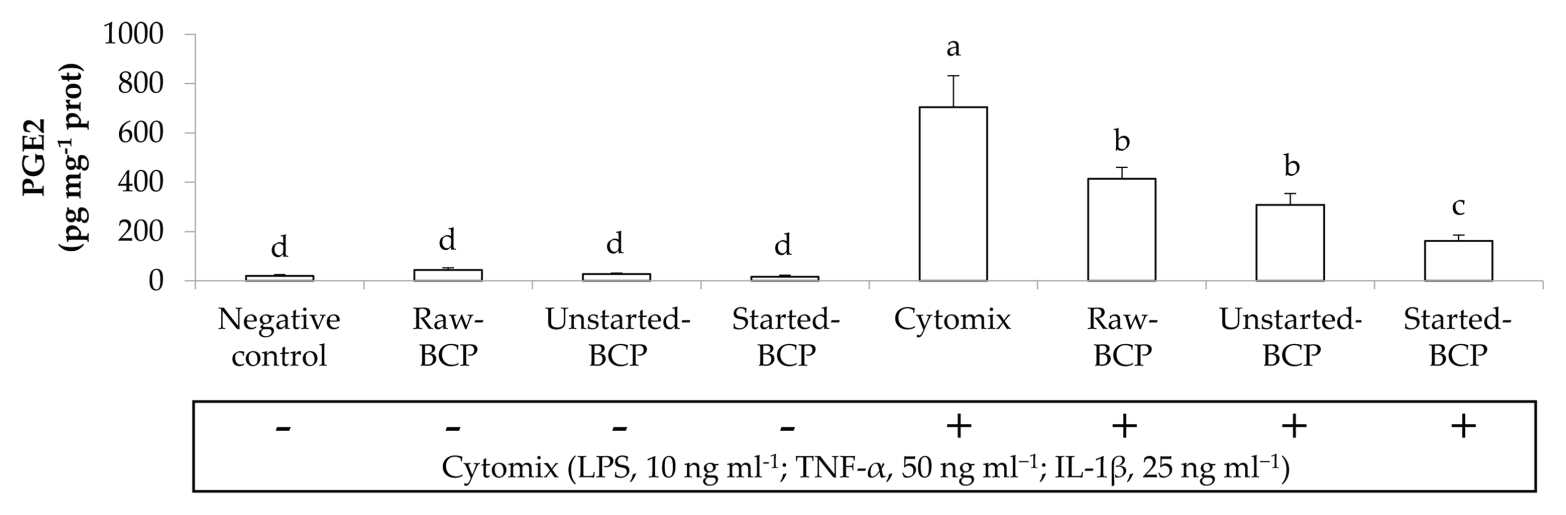
Prostaglandin E2 (PGE2) amounts (pg mg^−1^ prot) released by Caco-2 cells. Cells were treated for 24 h with 100 μg ml^−1^ of Raw-BCP, Unstarted-BCP, and Started-BCP. A set of samples was stimulated with the cytomix (LPS, 10 ng ml^−1^; TNF-α, 50 ng ml^−1^; and IL-1, 25 ng ml^−1^) in the last 18 h. Levels of PGE2 released by the untreated cells (negative control) and by cells treated only with the cytomix (positive control) were also reported. Data are the means (±SD) of three biological replicates analyzed in triplicate. Bars with different superscript letters are significantly different (*p* < 0.05).

Bee-collected pollen treatments did not significantly (*p* < 0.05) modified the secretion of MCP-1, TNF-α, and PGE2 by Caco-2 cells under regular physiological conditions, but strongly counteracted the pro-inflammatory stimulus induced by the IL-1β or the cytokines mix (Figures 2, 3). Overall, Started-BCP exterted the highest (*p* < 0.05) inhibitory effect, followed by Raw- and Unstarted-BCP.

### Intracellular Reactive Oxygen Species

**Figure 4:**
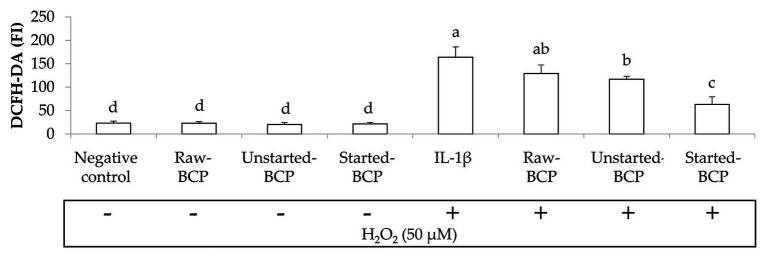
Intracellular reactive oxygen species (ROS) levels in Caco-2 cells, measured as the fluorescence intensity (FI) of 20,70-dichlorofluorescin diacetate (DCFH-DA). Caco-2 cells were treated for 24 h with 100 μg ml^−1^ of Raw-BCP, Unstarted-BCP, and Started-BCP. A set of samples was exposed to 50 μM H_2_O_2_ in the last 6 h to induce oxidative stress. Levels of ROS in the untreated cells (negative control) and in cells treated only with H_2_O_2_ (positive control) were also reported. Data are the means (± SD) of three biological replicates analyzed in triplicate. Bars with different superscript letters are significantly different (*p* < 0.05).

To gain insights into protective effects of BCP against oxidative stress, we investigated the accumulation of ROS within Caco-2 cells under the regular redox cell state as well under H_2_O_2_-induced oxidative stress. Intracellular ROS were measured by the probe 2',7'dichlorofluorescindiacetate (DCFH-DA) oxidation (Figure 4). BCP treatments did not induce any oxidative stress in Caco-2 cells. Intracellular ROS significantly (*p* < 0.05) increased when the cells were treated with H_2_O_2_ (164 ± 22 FI) compared to the negative control (23 ± 4 FI). Pretreatment with BCP significantly counteracted the increase of ROS, in spite of the oxidative stress induced by H_2_O_2_, with the lowest (*p* < 0.05) level of ROS (63 ± 16 FI) detectable with Started-BCP (Figure 4).

### Effect of BCP Treatment on the Intestinal Barrier Function

**Figure 5:**
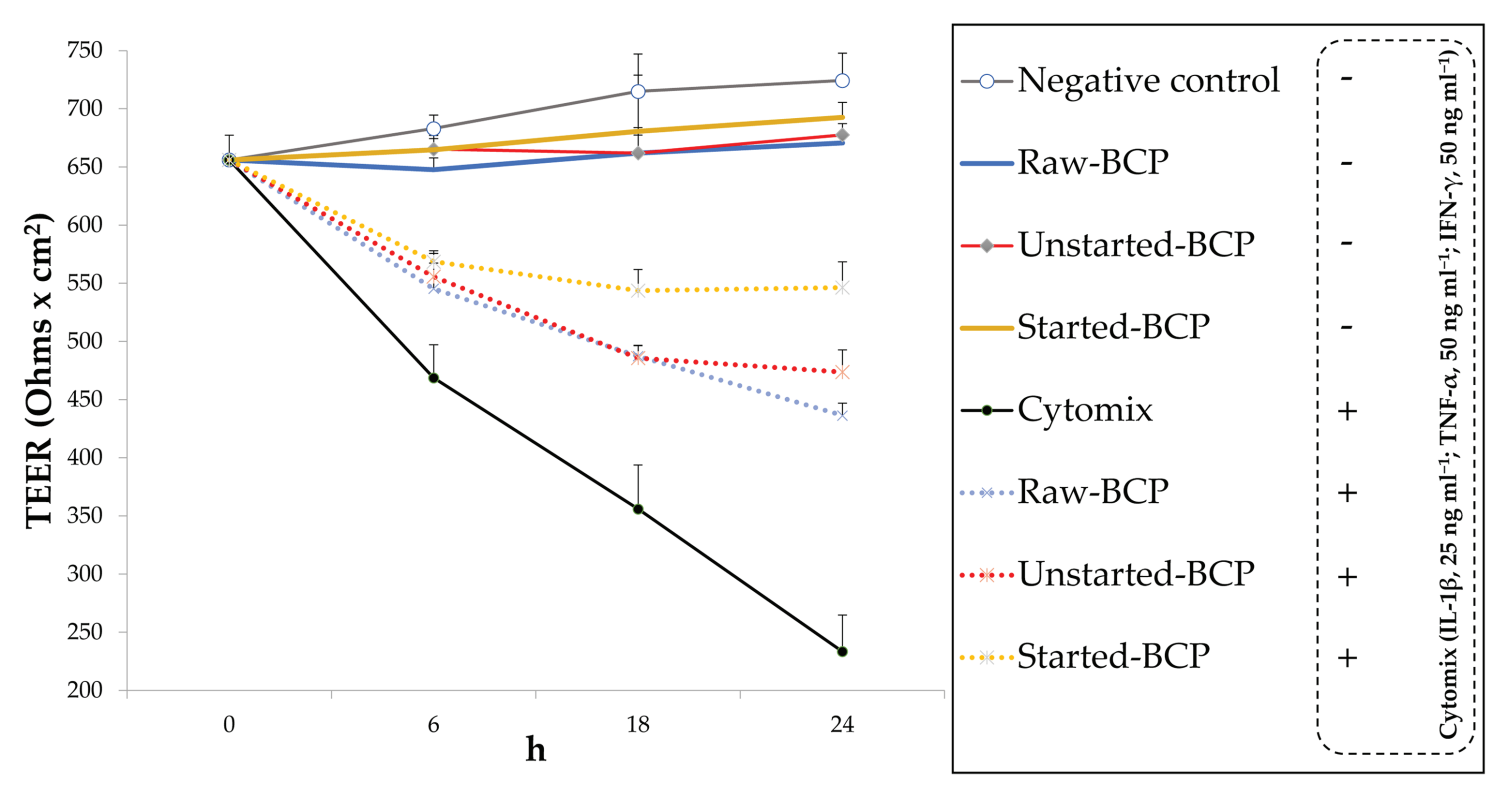
Transepithelial electric resistance (TEER; Ohms × cm^2^) of Caco-2 cells. Cells were treated for 24 h with 100 μg ml^−1^ of Raw-BCP, Unstarted-BCP, and Started-BCP. A set of samples was exposed to a mix of inflammatory cytokines (IL-1β, 25 ng ml^−1^; TNF-α, 50 ng ml^−1^; and IFN-γ, 50 ng ml^−1^) in the last 18 h. TEER levels of the untreated cells (negative control) and cells treated only with the cytomix (positive control) were also reported. Data are the means (± SD) of three biological replicates analyzed in triplicate.

The epithelial barrier function was monitored through the measurement of inflammation-induced changes of TEER and permeability in Caco-2 cells monolayers. Without a pro-inflammatory stimulus, BCP did not affect significantly (*p* > 0.05) the TEER with respect to the negative control (untreated cells; Figure 5). The exposure of Caco-2 cells to the mix of inflammatory cytokines induced a significant (*p* < 0.05) decrease of TEER (ca. 68%; Figure 5). Pretreatment of cells with BCP exerted a protective effect. After incubation with the pro-inflammatory stimulus, the lowest (*p* < 0.05) decrease of TEER was found with the Started-BCP treatments (ca. 26%; Figure 5). The protective effect was less effective when Caco-2 cells were treated with Raw- and Unstarted-BCP, leading to a decrease of TEER of ca. 43 and 37%, respectively (Figure 5).

**Figure 6:**
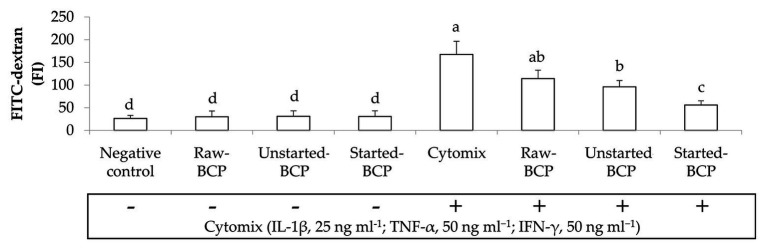
Fluorescence intensity of fluorescein isothiocyanate-dextran (FITC-dextran) permeated through the Caco-2 monolayers. Caco-2 cells were treated for 24 h with 100 μg ml^−1^ of Raw-BCP, Unstarted-BCP, and Started-BCP. A set of samples was exposed to a mix of inflammatory cytokines (IL-1β, 25 ng ml^−1^; TNF-α, 50 ng ml^−1^; and IFN-γ, 50 ng ml^−1^) in the last 18 h. Levels of FITC-dextran permeated through the untreated cells (negative control) and cells treated only with the cytomix (positive control) were also reported. Data are the means (± SD) of three biological replicates analyzed in triplicate. Bars with different superscript letters are significantly different (*p* < 0.05).

Accordingly to the changes of TEER, the exposure of Caco-2 cells to the pro-inflammatory cytomix significantly increased the permeability of cells monolayers (ca. 537%; Figure 6). BCP treatments significantly attenuated the inflammatory-induced permeability, with the lowest increase observed with the Started-BCP (ca. 113%), and followed by the Raw- and Unstarted-BCP treatments (ca. 334 and 265%, respectively; Figure 6).

### Anti-inflammatory Activity of BCP in Human Keratinocytes

**Figure 7:**
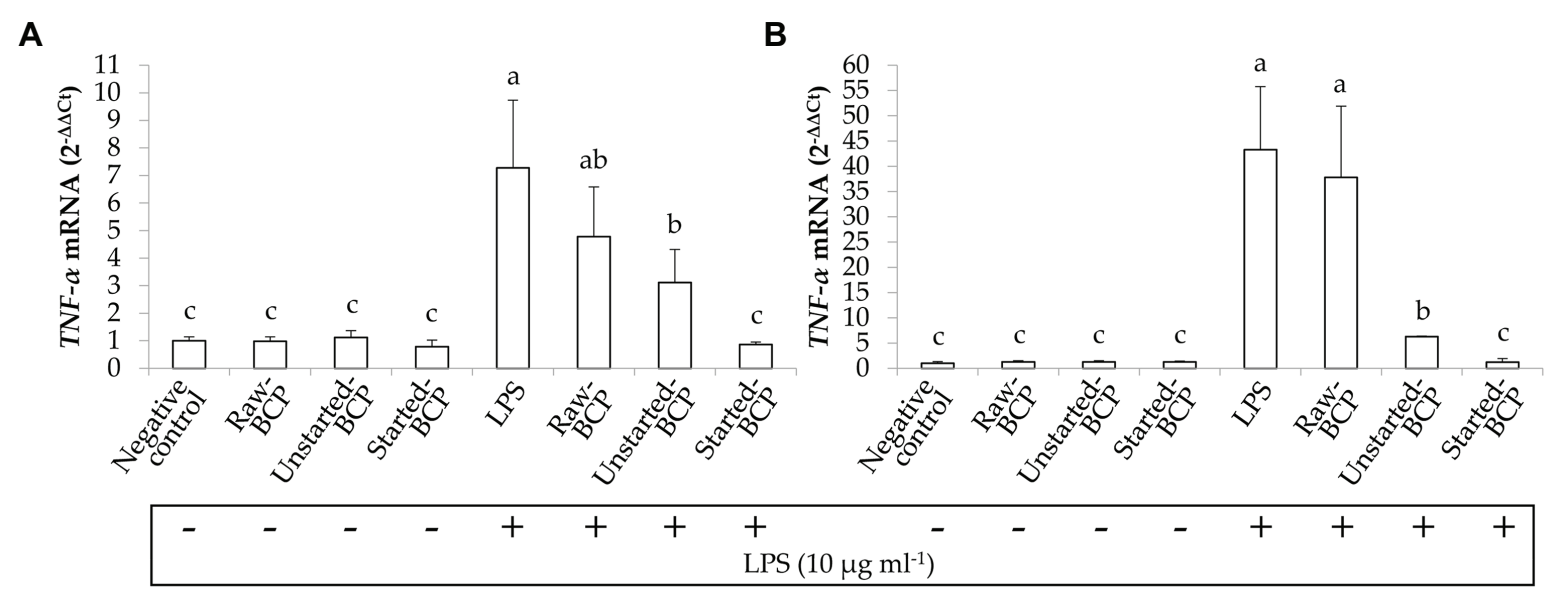
Relative expression (2^-*Δ*ΔCt^) of TNF-α gene in Caco-2 cells. Cells were treated for 16 **(A)** and 24 h **(B)** with Raw-BCP, Unstarted-BCP, and Started-BCP (100 μg ml^−1^). A set of samples were simultaneously exposed to LPS 10 μg ml^−1^. The relative expressions of TNF-α gene in the untreated cells (negative control) and cells treated only with LPS (positive control) were also reported. Data are the means (± SD) of three biological replicates analyzed in triplicate. Bars with different superscript letters are significantly different (*p* < 0.05).

Preliminarily, the cytotoxicity of BCP samples toward human keratinocytes was investigated through the MTT assay. BCP treatment up to 100 μg ml^−1^ showed no cytotoxic effects, thus a concentration of 100 μg ml^−1^ was selected for the subsequent assays on keratinocyte cells. The inflammatory response induced by LPS was evaluated by measuring the relative expression of TNF-*α* gene (Figure 7). Results showed that the levels of TNF-α mRNA were augmented by LPS-induced inflammation. Cells treatment with Started-BCP and, to a lesser extent, with Unstarted-BCP reduced (*p* < 0.05) the transcriptional levels of pro-inflammatory cytokine TNF-α (Figure 7).

## Discussion

Previous studies reported that BCP has a variety of valuable nutritional features and therapeutic effects (Khalifa et al., 2020; Thakur and Nanda, 2020), including those ameliorating the gut barrier function and the immune system within the gastrointestinal tract, the treatment of inflammatory status, and the prevention of oxidative stress damages (Chen et al., 2019; Li et al., 2019; Zhang et al., 2020). Beneficial effects of bioactive compounds from BCP depend on their bioaccessibility, which is defined as the amount released during digestion from the food matrix, and made available for small intestinal absorption. While a plethora of studies described the abundance of bioactive compounds in BCP, the outer layer of the grain pollen is not easily digestible by humans, resulting in a reduced nutrients bioaccessibility (Kieliszek et al., 2018; Zuluaga-Domínguez et al., 2019; Kostić et al., 2020). Furthermore, some bioactive compounds like phenolics are bound to the plant polysaccharides, which may hinder their release and solubilization in the chyme. To evaluate the nutrients and bioactive compounds bioaccessibility, we chose phenolics as target compounds. The highest serum-availability of phenolic compounds in Started-BCP suggests the positive effect of the fermentation on the nutrients bioaccessibility compared to Raw-BCP, likely due to the breakdown of pollen walls (Di Cagno et al., 2019a). Furthermore, the degradation of phenolics-associated proteins and carbohydrates by bacteria and yeasts likely contributed to the release of non-extractable phenolics (Tlais et al., 2021). Anyway, the increased bioaccessibility was found only under controlled fermentation conditions with selected starters, whereas the Unstarted-BCP did not differ with respect to Raw-BCP. These findings suggest that the microbial composition and the fermentation conditions represent key factors during BCP processing to enhance its available nutrients fraction for intestinal absorption (Di Cagno et al., 2019a). Once fermentation was showed to affect the bioaccessibility of nutrients and bioactive compounds, we further investigated whether fermented BCP might exert improved anti-inflammatory and immuno-modulatory features compared to Raw-BCP. The inhibition of acute phase inflammatory reaction represents one of the main targets for treatment of chronic inflammatory disorders of the gastrointestinal tract, such as irritable bowel syndrome (IBS; Sinagra et al., 2016; Di Cagno et al., 2019b). Many studies highlighted the key role of pro-inflammatory mediators, such as IL-8, IL-6, MCP-1, TNF-α, and PGE2, in the pathogenesis of inflammatory diseases (Wang et al., 2015). Under the conditions of our study, the treatment with Started-BCP halted the dramatic increase of IL-8, IL-6, MCP-1, TNF-α, and PGE2 levels induced by pro-inflammatory stimulus in Caco-2 cells. The same protective effect was negligible after treatments with Raw- or Unstarted-BCP. Other than for immunological studies, Caco-2 cells represent one of the most widely used models to mimic the intestinal mucosa, because they are able to spontaneously differentiate and form tight junctions, thus resembling normal intestinal epithelium. The increased permeability of intestinal epithelium and the impairment of its barrier function are involved in the pathogenesis of chronic inflammatory disorders of the gastrointestinal tract (Michielan and D’Incà, 2015; Landy et al., 2016). Through the addition of inflammatory stimuli, we experimentally induced tight junction dysregulation and intestinal barrier impairment in Caco-2 cells monolayers. As stated by the higher TEER value and lower permeability level, Started-BCP counteracted the deleterious effects on the integrity of the Caco-2 cells monolayer and its barrier function more effectively than found with Raw- and Unstarted-BCP. ROS have been recognized as mediators of inflammatory processes occurring in most of chronic inflammatory diseases, included IBS (Alzoghaibi, 2013; Pereira et al., 2017). Whether, under regular physiological conditions, a balance occurs between the production of ROS and the antioxidant enzymatic and non-enzymatic defense mechanisms, the oxidative stress appears in human body when the antioxidant defenses are overwhelmed by ROS accumulation, leading to the damage of cellular structures and biomacromolecules. Human’s endogenous antioxidant defense systems are complemented by dietary originating reducing compounds, such as vitamins and phenolics (Bouayed and Bohn, 2010; Halliwell et al., 2020). We investigated the protective effect of BCP samples against the oxidative stress, by using Caco-2 cell lines as model. Started-BCP successfully counteracted the H_2_O_2_-induced intracellular accumulation of ROS, whereas only a negligible effect was observed with the Unstarted-BCP. Several approaches may be pursued to enrich our body with exogenous bioactive compounds, other than through the diet, including dermatological applications (Cohen et al., 1991; Tebbe et al., 1997). Keratinocytes are active players in epidermal repair and in the skin’s immune defense through the secretion of growth factors and cytokines, and represent a suitable tool in mechanistic studies of inflammation and drug development (Pastore et al., 2011; Magcwebeba et al., 2016). We showed that Started-BCP may have a protective role against LPS-induced inflammation in human keratinocyte line HaCaT, counteracting the TNF-α accumulation. TNF-α is a pro-inflammatory cytokine that activates the endogenous inflammatory cascade, and it is responsible in chronic inflammation as periodontitis and psoriasis (Chiricozzi et al., 2011; Lagha and Grenier, 2019).

It is worth noting that phytochemicals may display harmful or beneficial effects on human cells depending by their dose-dependent behavior (Bouayed and Bohn, 2010). Treatments with BCP at a concentration of 100 μg ml^−1^ did not undermined the viability of Caco-2 and keratinocyte cells. Under regular physiological conditions and without any inflammatory stimulus, the treatment with BCP did not compromise the profile of inflammatory biomarkers and the intracellular ROS level in human cells, or the integrity of the Caco-2 cells monolayer, suggesting the absence of adverse effects of BCP.

The major findings of our study concern the improved anti-inflammatory and immuno-modulatory features of Started-BCP compared to Raw- and Unstarted-BCP. The beneficial functional properties of Started-BCP are likely attributable to the greater bioaccessibility of inherent bioactive compounds, like phenolics. BCP represents a valuable reservoir of phenolic compounds, especially flavonoids, phenolic acids, and phenolamines, which have been shown to scavenge free radicals and ROS, and protect against oxidative stress injury (Zhang et al., 2020). Independently of their antioxidant activity, phenolics are also able to interfer with inflammatory transduction pathways, by interacting with intracellular signaling cascades or by binding to the ATP-binding sites of several proteins (Bouayed and Bohn, 2010). Dietary phenolics have been also shown to modulate the expression of tight junction proteins in human intestinal cell monolayers (Bianchi et al., 2019). While it is widely recognized that BCP contains a plethora of essential nutrients and phytochemicals, several studies emphasized that Raw-BCP is not easily digestible, resulting in low bioaccessibility of its valuable costituents (Kieliszek et al., 2018; Zuluaga-Domínguez et al., 2019; Kostić et al., 2020). Other than to the increased nutrients bioaccessibility, the improved beneficial functions of Started-BCP might also be attributable to the microbial metabolites released during BCP fermentation (Filannino et al., 2018). For instance, microbial catabolites resulting from phenolics metabolism might have major biological and antioxidant activities compared to their precursors (Filannino et al., 2018). Others microbial derivatives, like alkyl catechols (e.g., 4-vinylcatechol, and 4-ethylcatechol), are able to interfere with the induction of NF-kB and MAPKs signaling pathways in mammalian cells (Filannino et al., 2018). The release of anti-inflammatory peptides (Saisavoey et al., 2020) due to the intense protein hydrolysys during BCP fermentation may not be excluded (Di Cagno et al., 2019a). Spontaneus fermentation occurring into the Unstarted-BCP produced negligible changes of BCP bioactivities compared to Started-BCP. The apparent inefficacy of spontaneous fermentations might be ascribed to the uncontrolled growth of yeasts, molds, and other bacterial groups and to the slower and less intense biochemical changes occurring into Started-BCP (Di Cagno et al., 2019a).

Fermentation of BCP with selected microbial starters provides a biotechnological solution to the issues concerning to the low digestibility of BCP by humans. Fermentation also allows to combine the unique profile of nutrients and bioactive compounds of BCP and the additional effect of microbial fermentation, improving its nutritional and functional features. The fermentation protocol, we studied results in BCP containing macro and micro-nutrients easier to be absorbed, which exert a beneficial role on inflammatory dysfunction through their effects on oxidative stress, inflammatory mediators, and pathways, and the intestinal barrier integrity in humans. We also highlighted how the effects of fermentation on BCP are strongly dependent by the use of selected microbial starters.

## Data Availability Statement

The original contributions presented in the study are included in the article/supplementary material, further inquiries can be directed to the corresponding authors.

## Author Contributions

PF, OV, DP, FM, and APr carried out the experiments. PF and RC conceived the study and administrated the project. PF, RC, OV, and APl elaborated the results and wrote the draft of the manuscript. MG was the scientific advisor and critically revised the manuscript. All authors contributed to the article and approved the submitted version.

### Conflict of Interest

DP was employed by the company Giuliani S.p.A (Milan, Italy).

The remaining authors declare that the research was conducted in the absence of any commercial or financial relationships that could be construed as a potential conflict of interest.
